# Clinical predictive artificial intelligence evaluation: A narrative review of trial designs and practical considerations

**DOI:** 10.1371/journal.pdig.0001621

**Published:** 2026-08-11

**Authors:** Maxime Fosset, Joris Pensier, Boris Jung, Arjumand Siddiqi, Muhammad Mamdani, Leo Anthony Celi

**Affiliations:** 1 Medical Intensive Care Unit, Lapeyronie Montpellier University Hospital, University of Montpellier, Montpellier, France; 2 Desbrest Institute of Epidemiology and Public Health, University of Montpellier, Montpellier, France; 3 Center for Anesthesia Research Excellence, Beth Israel Deaconess Medical Center, Harvard Medical School, Boston, Massachusetts, United States of America; 4 Department of Anesthesia, Critical Care and Pain Medicine, Beth Israel, Deaconess Medical Center, Harvard Medical School, Boston, Massachusetts, United States of America; 5 Anesthesiology and Intensive Care, Anesthesia and Critical Care Department B, Saint Eloi Teaching Hospital, PhyMedExp, University of Montpellier, INSERM U1046, Montpellier, France; 6 Department of Pulmonary, Critical Care and Sleep Medicine, Beth Israel Deaconess Medical Center, Harvard Medical School, Boston, Massachusetts, United States of America; 7 Medical Intensive Care Unit and PhyMedExp, Lapeyronie Teaching Hospital, University of Montpellier, Montpellier, France; 8 Dalla Lana School of Public Health, University of Toronto, Toronto, Ontario, Canada; 9 Edwin S.H. Leong Centre for Healthy Children, Hospital for Sick Children, Toronto, Ontario, Canada; 10 Department of Social and Behavioral Sciences, Harvard T.H. Chan School of Public Health, Boston, Massachusetts, United States of America; 11 Department of Health Behavior, Gillings School of Global Public Health, University of North Carolina at Chapel Hill, Chapel Hill, North Carolina, United States of America; 12 Data Science and Advanced Analytics, Unity Health Toronto, Toronto, Ontario, Canada; 13 Center for Artificial Intelligence Education and Research in Medicine, Temerty Faculty of Medicine, University of Toronto, Toronto, Ontario, Canada; 14 Laboratory for Computational Physiology, Massachusetts Institute of Technology, Cambridge, Massachusetts, United States of America; 15 Department of Biostatistics, Harvard T.H. Chan School of Public Health, Boston, Massachusetts, United States of America; University of Reading Reading School of Pharmacy, UNITED KINGDOM OF GREAT BRITAIN AND NORTHERN IRELAND

## Abstract

Artificial intelligence (AI) predictive models demonstrate potential for transforming clinical decision-making across medicine. However, conventional randomized controlled trials (RCTs), the gold standard for evaluating medical interventions, are ill-suited for clinical AI tools due to their static design, lengthy timelines, and inability to accommodate algorithms that evolve and adapt to changing clinical contexts. In this narrative review, we outline the limitations of traditional evaluation frameworks and propose a paradigm shift toward adaptive, iterative, and context-specific assessment methodologies. Evaluating clinical AI in practice requires three interdependent but epistemologically distinct activities: performance monitoring, which tracks the technical characteristics of the deployed model (calibration, discrimination, data drift, alert burden, fairness, workflow fidelity); clinical impact monitoring, which observationally and prospectively tracks whether the initial clinical benefit appears sustained over time; and scientific evidence generation, which produces causal estimates of deployment effects on patient outcomes through pragmatic, adaptive trial designs and causal inference techniques. We propose a predictive-AI-specific framework that links performance monitoring, clinical impact monitoring, evidence generation, causal estimands, and governance of model updates into one coherent decision pathway for clinicians and trialists. We present a governance-driven escalation protocol specifying when monitoring signals should trigger formal evidence generation, a decision pathway mapping signal types (performance or clinical impact) to trial design classifications, and a guide to causal inference methods for clinical AI trials. Drawing from adaptive platform and pragmatic trial designs, we recommend continuous monitoring approaches that prioritize patient-centered outcomes, health equity, and workflow integration over narrow performance metrics, and provide actionable steps to design a clinical AI trial. Successful implementation requires clinician engagement, transparency, and ongoing education regarding AI capabilities and limitations. Within this new evaluation paradigm, predictive AI can progress from a promising technology to reliable clinical tools that improve patient outcomes, support clinical decision-making, and uphold ethical standards in routine practice.

## Introduction

“After hearing for several decades that computers will soon be able to assist with difficult diagnoses, the practicing physician may well wonder why the revolution has not occurred. Skepticism at this point is understandable. Few, if any, programs currently have active roles as consultants to physicians. The story behind these unfulfilled expectations is instructive and, we believe, offers hope for the future [1].” This statement about the stale state of artificial intelligence (AI) in clinical medicine, despite being made four decades ago, rings true today.

In this narrative review, predictive AI denotes algorithms that generate risk scores or outcome predictions from clinical data; this differs from generative AI, such as large language models, whose primary function is to create new text or images rather than support bedside decision-making through probabilistic estimates [2]. Predictive AI holds tremendous promise for transforming clinical decision making, enhancing diagnostic accuracy, and improving patient outcomes. From chest X-ray interpretation [3] to mortality prediction from electrocardiograms [4] and sepsis treatment recommendations [5], predictive AI models have sparked hype for their potential to deliver real-time, multimodal data-driven insights. However, AI model drift, which pertains to the decay of performance over time as a result of data drift (changes in the distribution or statistical relationship of the variables), practice changes, or major events (such as the COVID-19 pandemic) among other factors, poses a fundamental challenge [6]. A clear illustration comes from the Epic Sepsis Model (ESM) [7], a widely deployed commercial sepsis prediction tool whose performance declined substantially over time as practice patterns, coding behaviors, and patient characteristics shifted. In a large independent evaluation, the ESM, which had been adopted across hundreds of U.S. hospitals, identified only 7% of sepsis cases it was designed to detect, while generating excessive false alerts due to calibration drift. This demonstrates how rapidly evolving data environments can render evaluation results obsolete by the time a trial is completed.

In addition, current benchmarks for model performance, a proxy for AI’s value, have rarely translated into enhancements in patient outcomes or clinical workflow efficiency when subjected to prospective evaluation [8]. While many clinical-AI tools have been evaluated via observational and real-world implementation studies to estimate their impact in routine care, the gold standard for causal inference and estimation of treatment effect of interventions remains randomized controlled trials (RCTs) [9]. However, the duration of an average RCT, from design and ethics approval to execution, analysis, and publication, often spans several years. By the time a model demonstrates a treatment effect, it may already be outdated, rendering the RCT’s conclusions less relevant. This slow cycle makes traditional trials poorly suited to evaluating AI systems with frequent updates.

In addition, ethical, workflow, and equity concerns frequently remain underexplored in static RCT designs [10]. AI tools must adapt to shifting data distributions, regulatory shifts, emerging clinical knowledge, and the consequent evolving practice, and hard-to-predict behavioral changes among its users, among others. These conditions are almost never accommodated in traditional RCTs, precisely because the goal of most RCTs is to control out or average across such conditions, to provide an overall estimate of efficacy. Researchers are therefore appropriately advocating for a new paradigm of iterative and adaptive, and more crucially, local and continuous evaluation of health AI.

Evaluating clinical AI in practice requires three interdependent but epistemologically distinct activities, each addressing a different inferential question.

**Performance monitoring** is the continuous operational surveillance of the technical characteristics of the deployed model, including calibration, discrimination, data drift, alert burden, fairness-related performance across subgroups, and fidelity to the intended workflow [11].

**Clinical impact monitoring** is the observational and prospective surveillance of clinical endpoints (such as mortality, length of stay, and adverse events) and fairness outcomes across the population exposed to the deployed model. It tracks whether the initial clinical benefit appears sustained over time but lacks a counterfactual comparator and therefore does not establish causal attribution to the AI tool. While valuable as a warning system, it generates a comparatively late signal, especially when the relevant outcomes are recorded over long timeframes, (i.e., long-term mortality), and cannot serve as the sole post-deployment monitoring strategy.

**Scientific evidence generation** is the formal investigation that produces causal estimates of AI deployment effects on patient outcomes through pragmatic, adaptive trial designs, evolving from randomized controlled trials and incorporating causal inference techniques.

It answers the question “Who benefits from the tool, under what conditions and through which mechanisms?” [12,13].

A deployed model may retain its technical characteristics, yet cease to deliver clinical benefit, for instance when clinicians modify their workflows over time so that alerts no longer translate into action. Conversely, a technical performance drift signal does not always imply a loss of clinical benefit, if clinicians override patterns of prediction that they judge not as useful as before.

This decoupling is the epistemological foundation that explains why performance monitoring cannot substitute for clinical impact monitoring and why clinical impact monitoring cannot substitute for scientific evidence generation when causal attribution is required.

Existing frameworks address these activities separately: quality-improvement frameworks specify monitoring protocols without defining when a formal trial should follow [14], while reporting guidelines govern trial conduct without linking it to the operational signals that motivated the evaluation. What remains unspecified is the structured interface between performance monitoring, clinical impact monitoring, and scientific evidence generation: the decision rules that determine when a monitoring signal should trigger formal re-evaluation or when clinical impact monitoring is sufficient, what causal estimands that evaluation should target, and which trial design is appropriate to the inferential question at hand.

In this narrative review, we aimed at synthesizing conceptual, methodological, regulatory, and practical considerations for the evaluation of predictive clinical AI, to propose a specific framework that links performance monitoring, clinical impact monitoring, hybrid evidence generation, causal estimands, and governance of model updates into one coherent decision pathway for clinicians and trialists.

We drew on established reporting guidelines (e.g., SPIRIT-AI, CONSORT-AI, DECIDE-AI, TRIPOD+AI, PROBAST+AI, STARD-AI), recent regulatory frameworks from the FDA and EU, main implementation research studies, as well as influential clinical studies and scoping reviews evaluating clinical AI systems.

We also discuss how to ensure that AI implementation leads to better care through community-directed selection of outcomes to measure as well as templates for evaluation, fairness considerations, and a reimagined trial design that prioritizes equity, safety, adaptability, and real-world efficacy, taking cues from adaptive platform trials and pragmatic trials.

## Current recommendations and landscape

In recent years, several guidelines and frameworks have emerged to guide the development, reporting, and oversight of clinical AI tools. The SPIRIT-AI [15] and CONSORT-AI [16] extensions outline requirements for protocol development and clinical trial reporting, respectively, ensuring that studies involving AI interventions specify how algorithms are integrated into clinical workflows and updated. The DECIDE-AI [17] guidelines focus on the early-stage, pre-deployment prediction models, clarifying how to document AI model updates. TRIPOD+AI [18] extends reporting recommendations for prediction model development and validation, while PROBAST+AI [19] offers a structured approach to assessing risk of bias for prediction models. The recently published STARD-AI guidelines [20] expand the original STARD checklist, ensuring transparent reporting of diagnostic accuracy studies involving AI. Table 1 summarizes the scope, intended use, and ideal application window for each of these guidelines in the lifecycle of an AI model.

**Table 1:** Comparison of major AI-related guidelines for clinical research. This table provides an overview of reporting and evaluation guidelines for clinical artificial intelligence, detailing their scope, stage of use, and application examples.

Guidelines	Primary scope	Stage of use	Key requirements	Example application
**SPIRIT-AI** (2020)	Protocol development for trials involving AI interventions	*Pre-trial*	Specify model versioning, update procedures, human oversight, data handling	Designing the protocol for a trial evaluating an AI-driven early warning system
**CONSORT-AI** (2020)	Reporting completed clinical trials involving AI	*Post-trial reporting*	Transparent reporting of intervention delivery, data flow, missing data, model updates	Writing the manuscript for an RCT testing AI-assisted CT interpretation
**DECIDE-AI** (2022)	Early-stage, pre-deployment evaluation of clinical AI systems	*Feasibility / pilot phase*	Document workflow integration, clinician–AI interaction, update iterations	Piloting a decision-support tool for antibiotic dosing in one clinical unit
**TRIPOD+AI** (2024)	Reporting prediction model development and validation	*Model development + external validation*	Detailed description of dataset, preprocessing, performance metrics, calibration	Describing a machine-learning mortality prediction model in manuscript form
**PROBAST+AI** (2025)	Risk of bias and applicability evaluation for prediction models	*Quality assessment*	Evaluate bias from participant selection, predictors, outcomes, and analysis	Appraising a hospital readmission prediction model for multi-site deployment
**STARD-AI** (2025)	Reporting of diagnostic accuracy studies involving AI-based tests.	*Diagnostic test evaluation (prospective or retrospective) and reporting.*	Extend the original STARD checklist to ensure clear description of the AI system (architecture, training data, version), across a broad range of diagnostic modalities (imaging, pathology, clinical data)	Reporting a multicenter diagnostic accuracy study comparing an AI model and radiologists for detection of pulmonary embolism on CT pulmonary angiography.

Beyond reporting frameworks, regulatory bodies and global organizations have issued high-level guidance. The World Health Organization (WHO) released updated recommendations on the ethics and governance of AI in health in 2023, emphasizing equity, transparency, and data governance, particularly in resource-limited settings [21]. The U.S. Food and Drug Administration (FDA) has also updated its action plan for AI/ML-based software, adopting a total product lifecycle approach that expects continuous local post-market monitoring, and formalizing the Predetermined Change Control Plan (PCCP) to accommodate safe ongoing updates of ML medical devices [22–24]. The STANDING Together consensus [25] establishes recommendations for addressing algorithmic bias at the foundational level of AI models: data. It notably recommends identifying disparate performance among groups of users to ensure fairness. Further work is warranted to determine the most effective and practical approach for conducting these bias analyses.

These recommendations repeatedly underscore the importance of real-world validation, model performance transparency, and careful attention to fairness metrics. However, little consideration is made by these efforts to confront the fact that the static evaluations that are at the center of these initiatives fail to capture the iterative refinements necessary for AI systems that learn from continuous new data. On paper, AI tools have shown promising results in retrospective analyses or tightly controlled feasibility studies. But they have struggled to demonstrate real-world impact when rigidly evaluated under classic RCT constraints [8,26,27]. The frameworks in this review focus on predictive AI, which uses algorithms to generate risk scores or outcome probabilities from structured clinical data. Large language models (LLMs) deployed in clinical decision support need a different evaluation approach, outlined in the TRIPOD-LLM guidelines [28]. The stochastic free-text outputs of LLMs that vary across formally identical queries might preclude from precise intervention specification in trials protocols, and raise novel challenges in equity assessment as bias might emerge from language patterns that can change clinical reasoning [29]. Rosenthal et al. [30] have recently proposed a dynamic deployment framework addressing the specific challenges of LLM clinical trials, including continuous in-situ adaptation and real-time monitoring of evolving model behavior. Nonetheless, the governance and causal inference frameworks described in the present review represent necessary but insufficient foundations for LLM evaluation.

## Why traditional RCTs may not work for AI

RCTs are widely accepted as the gold standard for assessing the efficacy of medical interventions [31]. However, the peculiarities of AI as described above render static study designs less suitable for several reasons.

First, AI models evolve rapidly. Algorithms often require updates to accommodate new data distributions, shifting clinical practice and consequent behavioral change of the user, whether a clinician or a patient. An RCT that typically takes at least a year or more to complete may conclude with results that are no longer relevant as the model is already outdated at the conclusion of the study [32]. This risk is best illustrated by the transition in sepsis management. For years, AI models such as the Epic Sepsis Model were trained and evaluated using “Sepsis-2” (SIRS-based) criteria. When international guidelines shifted to “Sepsis-3” (SOFA-based organ dysfunction) in 2016, the collectively adopted ground truth for sepsis effectively changed. Validation studies have since shown that models trained on the old definition perform erratically when evaluated against the new standard, as they are essentially predicting a phenotype that clinical consensus has discarded [33]. An RCT spanning such a guideline shift would face catastrophic confounding, as the control arm (standard of care) and the outcome definition would fundamentally change during the study.

Second, AI learns through continuous feedback. Predictive systems, particularly those based on machine learning or reinforcement learning, recalibrate in near-real time to new data streams [2,34]. By contrast, traditional RCT designs are fixed at the outset, making them a poor fit for technologies that rely on ongoing recalibration. As a result, traditional RCTs are more frequently applied to machine learning models without recalibration, which may hinder the effectiveness of AI models. Furthermore, traditional RCT designs rely on the assumption of intervention stability, specifically that the tool itself does not alter the underlying data generation process. However, AI deployment frequently induces performative prediction, a phenomenon in which the model’s output influences clinical decision-making, thereby shifting the distribution of future data and invalidating the model’s baseline training [35].

For instance, a sepsis prediction algorithm may prompt earlier prophylactic treatment, resulting in improved patient survival. Consequently, these high-risk patients generate better outcome data. If the model is retrained on this post-intervention data, it may erroneously learn to associate high-risk phenotypes with low-risk outcomes, a degradation of predictive utility known as feedback loop failure [36].

Third, contrary to widely held notion, the generalizability of effect estimates derived in clinical trials is limited in time and space. The treatment effect that is provided by a study requires that the patient demographic and the practice patterns are reflective of the patient and practice characteristics of each clinical entity applying their results, an extremely tall feat. AI predictive models take on, rather than avoid, contextual factors. AI performance therefore depends heavily on the “context” of the data, the patient demographics, the data collection processes, and the clinical workflows, among others. Evaluating AI in a single or even multiple sites will not predict its performance across diverse settings, particularly in critical access hospitals and those in low- and middle-income countries (LMICs) [37]. Moreover, the argument of the predictive methodology is that it should not attempt to predict across these settings, but rather should embrace their specifics by incorporating contextual data in order to create a more accurate model for any given setting.

For example, a comprehensive study assessed clinical prediction model for the case study of antipsychotic treatments for schizophrenia across numerous hospitals and determined that their performance does not generalize effectively. A model trained at Hospital A demonstrates high performance within that setting; however, when applied to Hospital B, its accuracy diminishes to nearly random levels. This finding indicates that a successful single center RCT does not constitute evidence of widespread clinical applicability [38]. Fourth, artificial comparisons, such as AI versus clinicians or even against “human in the loop” systems are often reductive and not representative of typically hurried clinical decision-making [39]. As workflows vary wildly between hospitals, it is crucial to design and evaluate human-AI systems locally and repeatedly in anticipation of changes in clinician behavior once AI is incorporated in their workflows [40]. Trials focusing on evaluating behavioral changes after AI implementation are critical to AI deployment success.

RCTs evaluating the performance of AI in comparison to clinicians may overlook biases inherent to the interaction between AI and clinicians, such as automation bias. This bias involves the inappropriate acceptance of an inaccurate AI prediction solely due to its automated presentation, potentially resulting in a failure to identify errors or an excessive reliance on AI models [41].

Finally, while RCTs typically control environmental variables, real-world effectiveness is often dictated by sociotechnical factors such as user trust, hardware limitations, and workflow integration. For instance, a deep learning system for diabetic retinopathy, which demonstrated specialist-level accuracy in a large-scale retrospective study, failed during field deployment in Thailand. The failure was not algorithmic but environmental: poor lighting conditions and limited internet bandwidth disrupted nursing workflows, causing the system to reject images or time out [42]. This case underscores that trials should consider the “last mile” implementation challenges that determine clinical success.

## Adaptive and iterative approaches to clinical AI evaluation

**Fig 1:**
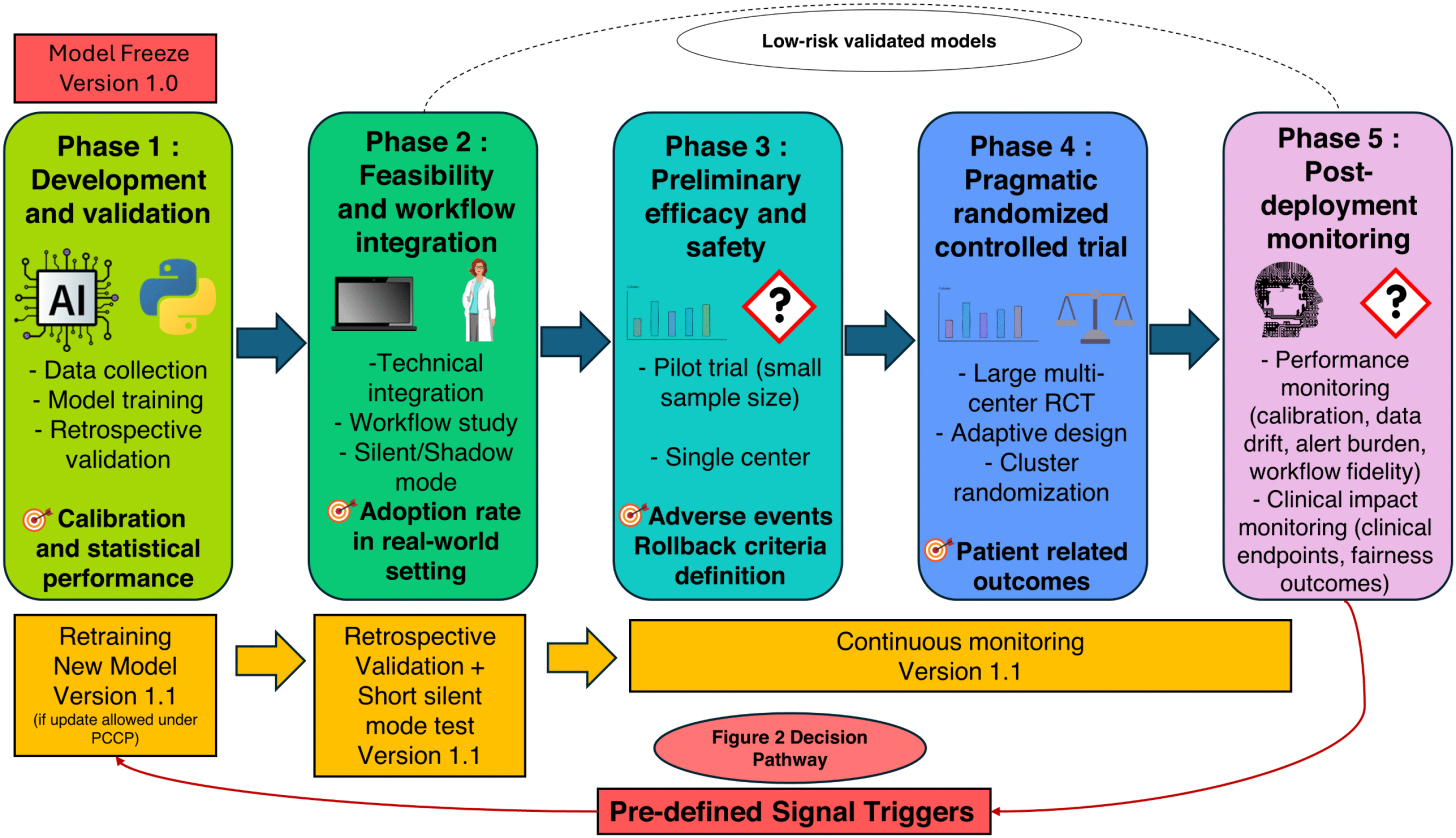
Iterative lifecycle for clinical AI evaluation. Lifecycle framework for clinical AI evaluation across five phases. Phase 1: model development and retrospective validation. Phase 2: feasibility and workflow integration. Phase 3: preliminary efficacy and safety (single-center pilot trial with adverse-event monitoring and pre-defined rollback criteria). Phase 4: pragmatic randomized controlled trial (multi-center, adaptive design, patient-related outcomes). Phase 5: post-deployment monitoring, encompassing performance monitoring (calibration, discrimination, data drift, alert burden, fairness across subgroups, workflow fidelity) and clinical impact monitoring (clinical endpoints, fairness outcomes). Solid arrows indicate the principal pathway through all five phases; the dotted arrow indicates the alternative pathway available for low-risk, externally validated models with stable operating characteristics, which may proceed from Phase 2 directly to Phase 5, omitting the trial phases (Phases 3–4) that are particularly indicated for novel or high-risk AI applications prior to clinical deployment. Pre-defined signal triggers detected during Phase 5 may invoke either a return to Phase 1 for retraining and safe redeployment of a new model version (e.g., v1.1, under PCCP constraints, with abbreviated retrospective validation and silent-mode testing) or the escalation pathway specified in Fig 2 when interpretation of the signal requires causal inference rather than continued observational surveillance. The lifecycle aligns with contemporary regulatory expectations (e.g., FDA PCCP, EU AI Act).

This section addresses two complementary methodological needs for clinical AI evaluation. The first is scientific evidence generation: designing trials that produce causal estimates of AI effects on patient outcomes. This should address not only whether the system works, but for whom, under what conditions, and with what effect size. The second is implementation governance: establishing the operational infrastructure that monitors deployed models and determines when a formal re-evaluation is warranted. We address evidence generation first, through adaptive platform trials and pragmatic trial designs, then describe the governance framework that connects operational surveillance to trial initiation (Table 2, Fig 1).

**Table 2:** Framework for a Governance-Driven Update Cycle.

Governance component	Description	Purpose	Example (Sepsis Model)
**Predetermined change boundaries**	Defines the scope of permissible updates (e.g., recalibration) vs. those requiring regulatory re-submission.	Ensures updates occur only within pre-approved limits.	Minor recalibration permitted; major architecture change prohibited during trial.
**Version control & documentation**	Centralized tracking of model versions, training data snapshots, features, hyperparameters, and release notes.	Ensures full traceability and reproducibility of model behavior.	Model v1.1 replaces v1.0 after drift correction, both archived.
**Pre-deployment validation**	Mandatory testing protocol prior to releasing an update (e.g., retrospective validation, silent-mode testing, fairness audits).	Prevents performance degradation or bias from entering clinical workflow.	7-day silent-mode test confirms stability before deployment.
**Post-deployment monitoring**	Continuous surveillance of performance, safety, and fairness metrics following an update.	Detects unintended consequences early.	Weekly calibration plots show restored accuracy.
**Pause/rollback criteria**	Objective triggers for temporarily disabling the model or reverting to earlier version.	Protects patient safety in real time.	Automatic Pause: Alerts disabled for patients age ≥ 70 upon drift detection.
**AI-QI oversight team**	Multidisciplinary group (clinicians, engineers, statisticians) responsible for continuous monitoring and transparent communication.	Ensures operational accountability and clinical context validation.	AI-QI team meets weekly to review dashboards.
**Regulatory alignment**	Conformity with FDA PCCP and EU AI Act requirements for lifecycle risk management.	Maintains legal compliance and traceable audit trails.	Update classified as “minor change” under PCCP.

**Fig 2:**
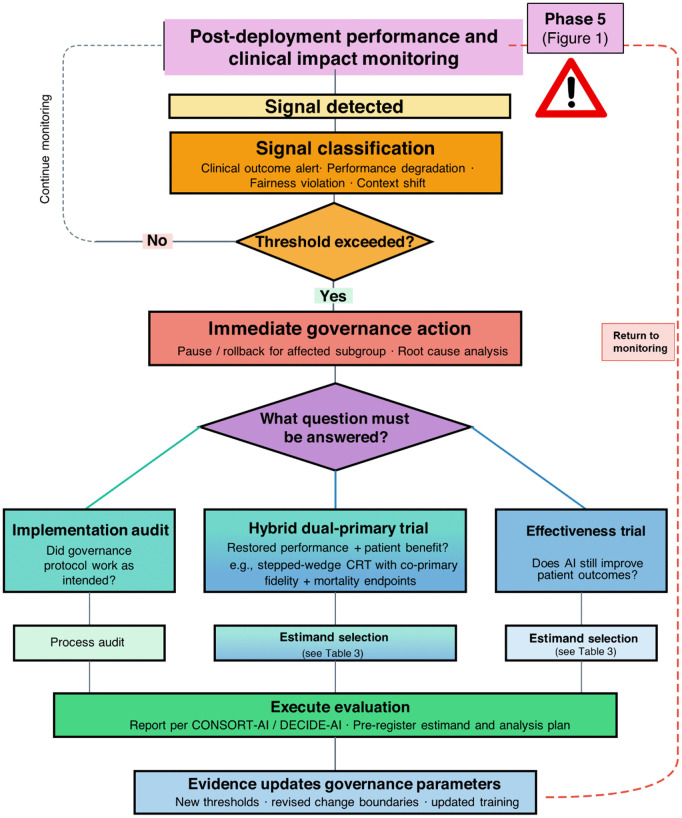
Decision pathway for escalating from performance and clinical impact monitoring to scientific evidence generation in clinical predictive AI evaluation. Post-deployment escalation pathway, detailing the response to a monitoring signal detected during Phase 5 of Fig 1 (post-deployment performance and clinical impact monitoring; the magenta header identifies this anchor and matches the Phase 5 coding in Fig 1). When a candidate signal is detected, the AI-QI team (jointly defined by the clinical domain expert, biostatistician, and regulatory affairs specialist) classifies it into one of four categories: clinical outcome alert, performance degradation, fairness violation, or context shift. If the signal does not exceed pre-specified thresholds (defined in the trial protocol using criteria such as calibration decline beyond a pre-defined margin, subgroup performance below a safety boundary, or fairness metrics breaching criteria defined with patient and community representatives), surveillance continues without escalation. If the threshold is exceeded, an immediate governance action is taken—pause or rollback for the affected subgroup, with root cause analysis—and the AI-QI team determines the inferential question that must be answered. Three pathways follow. An implementation audit assesses whether the governance protocol functioned as intended when the question concerns process fidelity. A hybrid dual-primary trial (for example, a stepped-wedge cluster randomized trial with co-primary endpoints combining model fidelity and a patient outcome such as mortality) is initiated when both restored model performance and continued clinical benefit must be re-established. An effectiveness trial is initiated when fundamental clinical effectiveness is in question; in this case, the deployment is paused pending re-evaluation. Estimand selection for the two trial designs follows Table 3. Evaluations are executed and reported following CONSORT-AI and DECIDE-AI, with pre-registered estimands and analysis plans. The generated evidence updates governance parameters (new thresholds, revised change boundaries, updated training) and feeds back into post-deployment monitoring (dashed red return arrow), closing the governance loop. This pathway does not duplicate the pre-deployment trial of Fig 1 (Phases 3–4); it defines when and how a deployed model should re-enter formal evaluation, which can use causal inference techniques and adaptive trial designs to avoid the cumbersome design of randomized controlled trials. Dashed arrows indicate return loops.

Adaptive platform trials use a single master protocol to evaluate multiple interventions simultaneously or sequentially, with prespecified rules allowing the addition, removal, or modification of treatment arms based on interim analyses. Unlike traditional fixed-design RCTs, adaptive platform trials continuously update randomization probabilities, sample size requirements, and intervention eligibility as emerging data accumulate, enabling faster learning and more efficient evidence generation [43]. This architecture maps naturally to clinical AI evaluation, because AI models often undergo ongoing updates (such as recalibration or threshold adjustments) based on performance drift or contextual changes. A platform-style structure allows new AI model versions to be incorporated as additional “arms,” older versions to be retired when no longer relevant, and safety signals to be assessed repeatedly across predefined patient subgroups. In contrast to fixed RCTs, adaptive platform trials explicitly anticipate iteration, making them better suited to technologies whose performance evolves quickly over time. Regarding this iterative aspect, modern clinical AI systems require explicit governance structures to manage model updates safely over time. Regulatory frameworks such as the FDA’s Predetermined Change Control Plan (PCCP) [24] for machine-learning medical devices and the European Union AI Act for high-risk health AI [44] now emphasize the need for pre-specified change boundaries, documentation of intended updates, and continuous post-deployment oversight. To operationalize these principles in clinical evaluation, AI trials should incorporate an “update governance” plan that outlines: (1) which components of the model may change (e.g., thresholds, features, risk calibration), (2) how version control and audit trails will be maintained, (3) what validation checks must occur before and after each update, (4) predefined criteria for pausing or rolling back a model if harm signals emerge, and (5) the responsible stakeholders, including data scientists, clinicians, safety officers through interdisciplinary AI quality-improvement (AI-QI) teams [14]. AI-QI oversight requires five core roles: a clinical domain expert who contextualizes performance alerts against bedside realities and identifies clinically meaningful drift; a data scientist or ML engineer responsible for model versioning, calibration monitoring, and updates within predetermined change boundaries; a biostatistician or clinical epidemiologist who defines estimands, specifies causal analysis plans, and determines when monitoring signals warrant escalation to formal evaluation (Fig 2); a regulatory affairs specialist who classifies updates under FDA PCCP or EU AI Act requirements and maintains audit trails; and a patient or community representative who ensures equity monitoring reflects the priorities of the populations served. Embedding such governance structures into adaptive and/or pragmatic trials enables safe iterative model refinement in ways that traditional fixed RCT designs cannot accommodate. This approach aligns with agile software development in technology; however, it demands a level of regulatory flexibility that many existing frameworks only partially accommodate.

To illustrate, consider the case of sepsis, where traditional RCTs face significant challenges including heterogeneous patient populations (source of infection, nature of infectious agent…), rapidly evolving clinical conditions, and time-sensitive intervention requirements that often yield non-generalizable results and require extended timeframes for completion. An AI-driven sepsis algorithm could be updated in real time for improved mortality prediction, and the adaptive trial infrastructure would methodically integrate the updated version. If a signal suggests harm to a specific patient cohort during periodic analysis, the algorithm is discontinued swiftly for that subgroup, prioritizing patient safety while allowing its use to continue in other cohorts. The subgroup for which the AI tool is halted reverts to standard care without the algorithm, and the algorithm is retrained on the new data that is collected while the tool is on hold. Once the model is recalibrated, it is re-deployed and the trial resumes for that patient subgroup with the appropriate statistical analysis. Similar to a platform trial, the study is particularly designed for a treatment effect that changes over time across patient subgroups and which requires continuous monitoring. But in contrast to a platform trial, the intervention is subject to periodic recalibration among subgroups whose outcomes are drifting away from the rest of the population. This design values AI fairness over average treatment effect. A case example of a governance-driven update cycle for this sepsis prediction model is shown in Table 2.

To understand how these governance components interact in practice, consider a sepsis model deployed under a PCCP-aligned plan. Three months post-deployment, the AI-QI Oversight Team detects drift (calibration deterioration in adults ≥70 years). The following cycle is triggered:

1. Pause (Safety First): Per Pause/Rollback Criteria, alerts are automatically disabled for the affected subgroup (age ≥ 70) while standard care resumes.
2. Evaluate: Root cause analysis reveals workflow changes altered input timing.
3. Update: Engineers implement a recalibration, checked against Predetermined Change Boundaries to ensure it qualifies as a minor, permitted update.
4. Validate: The new version (v1.1) undergoes Pre-Deployment Validation, including fairness checks and a 7-day silent-mode test.
5. Redeploy: Upon passing validation, v1.1 replaces v1.0. Post-Update Monitoring confirms calibration is restored.

The proposed framework defines a structured interface between operational signals and clinical re-evaluation rather than a mandatory sequence. Three pathways to evidence generation can coexist in the clinical AI lifecycle. First, **pre-deployment trials** (Fig 1, Phases 3 and 4) remain the appropriate path for novel or high-risk models whose clinical benefit has not been established; the trial is contemporary to deployment and is not triggered by a monitoring signal. Second, **monitoring-only deployments** are appropriate for low-risk, externally validated models with stable operating characteristics, where performance monitoring may continue indefinitely without escalating to formal evaluation; this should not be used as a surrogate for scientific evidence generation and should be reserved for workflow and human-AI cooperation studies in order to avoid exposing patients to harmful algorithms. Third, the **escalation pathway** described below applies when post-deployment monitoring detects a signal whose interpretation requires causal inference rather than further observational surveillance. Projects intended purely as a trial or purely as a monitored implementation are accommodated within the framework as the first or second pathway respectively.

The governance cycle described above specifies how deployed models are monitored and maintained. However, monitoring alone does not constitute scientific evidence generation. A critical operational question remains: when should a monitoring signal trigger a formal trial, and what kind of trial should follow? Recent frameworks have addressed adjacent challenges: You et al. proposed a clinical trials–informed four-phase implementation framework for AI deployment (safety, efficacy, effectiveness, monitoring) but did not classify each phase by its inferential goal or specify the causal estimands governing transitions between phases [45]; Rosenthal et al. introduced a dynamic deployment framework for large language model clinical trials with continuous in-situ adaptation and real-time monitoring, addressing a distinct evaluation context from the discrete-version predictive AI systems considered here [30]; and Matson et al. applied hybrid effectiveness-implementation design principles to digital behavioral health interventions, though not to predictive AI with its specific challenges of autonomous model updating, regulatory classification as software as a medical device, and calibration-dependent performance degradation [46]. The present framework addresses this gap by specifying the structured decision pathway linking monitoring signals to trial design selection and estimand determination (Fig 2). This escalation pathway operates on signals from both performance monitoring and clinical impact monitoring introduced above; pre-deployment trials for novel or high-risk AI follow Phases 3 and 4 of Fig 1 and are not triggered by a monitoring signal. When post-deployment surveillance detects calibration drift within pre-specified bounds and without measurable impact on patient outcomes, continued clinical monitoring under the governance protocol is sufficient. When performance degradation is detected with potential patient impact (for instance, declining predictive power in a vulnerable subgroup) a formal re-evaluation using a hybrid design [12] with co-primary implementation and patient outcome endpoints is warranted (for example, a stepped-wedge cluster randomized trial evaluating both whether the corrected model restores calibration fidelity and whether continued deployment maintains the intended mortality benefit). When a major failure occurs or an external context shift fundamentally alters the clinical environment (such as a change in disease definition or standard-of-care guidelines), the system should be paused, and a full effectiveness trial powered for patient outcomes should be initiated before re-deployment. In each scenario, the monitoring signal determines not only whether a trial is needed, but what causal estimand it should target — a specification provided in Table 3.

**Table 3:** Primary causal strategies for evaluating deployed predictive AI in clinical trials.

AI-Specific problem	Statistical challenge	Causal inference method	Formal estimand (ICH E9(R1))	Clinical AI example (Sepsis Prediction Model SPM)	Contraindication
**Time-varying effect estimation failure:** AI alerts alter clinician behavior, creating time-varying treatment–confounder feedback.	Past treatment (response to alert) affects future confounders (vital signs). Standard regression conditioning on post-treatment variables introduces collider bias.	Marginal structural models / inverse probability weighting (MSMs/IPW). Reweights observations to create a pseudo-population in which treatment is independent of time-varying confounders.	**Population:** ICU patients exposed to sepsis alerts **Treatment:** sustained AI-guided care A vs. no AI protocol B. **Outcome:** 30-day mortality. **Intercurrent event:** alert overrides, handled with a treatment-policy strategy. **Summary:** marginal contrast in 30-day mortality under sustained strategies.	SPM generates an alert, and the clinician initiates early antibiotics. This response may improve survival while also altering subsequent clinical trajectories and future training data. MSM/IPW estimates 30-day mortality under sustained AI-guided management versus usual care while accounting for alert-response feedback.	Not appropriate when positivity is violated, treatment strategies are poorly defined, or weights are unstable because of near-deterministic treatment patterns. Extreme weights require stabilization or truncation.
**Version stability failure:** the intervention changes during evaluation because the model is updated mid-trial.	Same patient cannot be observed under both versions. Standard before-after comparisons fail because the same patient cannot be observed under both model versions, and secular trends may be confounded with the version change.	Parametric g-formula. Models the joint distribution of time-varying covariates and outcomes; simulates counterfactual trajectories under each deployment regime.	**Population:** all patients evaluated during the deployment period. **Treatment:** continuous deployment of model version 1.0 versus the observed versioning sequence (v1.0 → v1.1). **Outcome:** 30-day mortality. **Intercurrent event:** model update, handled using a hypothetical strategy. **Summary:** difference in mean potential outcomes under alternative versioning regimes.	SPM v1.0 is deployed in January and recalibrated to v1.1 in June after performance drift is detected in older patients. The g-formula estimates 30-day mortality under uninterrupted v1.0 deployment versus the observed update sequence, adjusting for concurrent changes in patient acuity.	Not appropriate when the parametric models in the joint distribution are poorly specified, or when the number of time-varying covariates is too large relative to sample size.
**Subgroup identifiability failure:** the trial alone does not adequately identify the causal effect in a clinically important underrepresented subgroup.	Trial data alone are insufficient because subgroup sample size, covariate support, or event counts are too limited to estimate the subgroup-specific effect reliably.	Data fusion / evidence synthesis for subgroup effect estimation. Combines trial and observational data under a shared causal model to estimate subgroup-specific effects.	**Population:** patients aged over 75 years across all relevant evidence sources. **Treatment:** AI-guided care versus standard care. **Outcome:** 30-day mortality. **Intercurrent event:** site-level implementation heterogeneity, handled through stratification or modeling. **Summary:** subgroup-specific causal effect (CATE) in the target older patients subgroup.	SPM trial at academic center enrolls 12% older patients. Two community hospitals have observational SPM data with 35% older patients. Fusion combines trial data with observational deployment data to estimate the older patients-specific effect.	Not appropriate when the subgroup is absent across all sources, when subgroup definitions are inconsistent, or when exchangeability across data sources is implausible.
**Target-site transport failure:** the trial effect is identified in the study population but may not apply to the intended deployment setting.	Internal validity may hold in the trial population, but external validity fails because differences in case-mix, workflow, or care delivery may modify the treatment effect.	Transportability analysis/Target-population reweighting. Reweights trial estimates using target site’s covariate distribution under formalized transportability assumptions.	**Population:** patients at the target deployment hospital. **Treatment:** AI-guided care versus standard care. **Outcome:** 30-day mortality. **Intercurrent event:** workflow differences between study and target sites, handled using a treatment-policy strategy. **Summary:** average treatment effect transported to the target deployment setting.	SPM is evaluated in a tertiary hospital with high nurse-to-patient ratios but intended for deployment in a community hospital with different case-mix and workflows. Transportability reweighting uses the target hospital’s covariate distribution to estimate the expected local clinical benefit.	Not appropriate when key effect modifiers are unmeasured, when target-site covariates are unavailable, or when there is poor covariate overlap between source and target populations.

This table presents four causal inference methods matched to inferential challenges specific to deployed predictive AI. Each row specifies the AI-specific problem, the statistical challenge that standard analysis cannot address, the causal inference method and its mechanism, a formal estimand structured following ICH E9(R1) principles (population, treatment condition, variable, intercurrent event handling strategy, summary measure), a concrete application using for instance a sepsis prediction model, and conditions under which the method should not be applied. The intercurrent event handling strategies reference the ICH E9(R1) addendum classification: treatment-policy (observe all outcomes regardless of intercurrent event), hypothetical (estimate outcome in absence of intercurrent event), and principal stratum (restrict to subpopulation defined by intercurrent event behavior). Methodological anchors include marginal structural models for time-varying confounding [52], data-fusion/transportability theory [55], and target-population transport methods [57,58].

MSMs = marginal structural models; IPW = inverse probability weighting; CATE = conditional average treatment effect; ATE = average treatment effect; SPM = Sepsis Prediction Model

Governance framework for performance monitoring of deployed predictive AI. This framework addresses the operational surveillance of deployed models, such as tracking calibration, managing updates within regulatory boundaries, and ensuring governance protocol fidelity. It produces implementation outcomes (deployment fidelity, version compliance, alert burden management) that are necessary preconditions for, but do not substitute for, clinical impact monitoring or formal evidence of patient outcome benefit [11]. The governance signals generated by this monitoring process determine when formal scientific evidence generation should be initiated with a proposed decision pathway specified in Fig 2.

## Pragmatic trials for clinical AI

When the escalation pathway indicates that formal evidence generation is warranted, the choice of trial design must balance causal rigor with the practical constraints of evaluating an AI tool already embedded in clinical workflows.

Pragmatic trials [47], designed to evaluate interventions under real-world conditions rather than tightly controlled environments, offer a promising framework for clinical AI evaluation. Unlike explanatory trials that emphasize internal validity through strict inclusion/exclusion criteria, pragmatic trials prioritize external validity and generalizability by incorporating diverse patient populations and heterogeneous care settings. Pragmatic evaluations are particularly well-suited to technologies whose effects depend on clinical workflows, human–technology interaction, and local implementation contexts, making them a natural fit for clinical AI trials. When adapted for clinical AI, pragmatic trials could address the dynamic nature of AI algorithms by embedding evaluation within routine clinical workflows, allowing assessment of both performance drift and implementation challenges across diverse healthcare contexts. A key strength is their ability to capture clinician-AI interactions as they naturally evolve, rather than under artificial research conditions, potentially revealing unexpected workflow adaptations and decision-making patterns. This is particularly important for AI models because performance is directly shaped by user behavior, adherence, alert responsiveness, and workflow variation, factors that traditional explanatory trials often cannot capture. This can help solve the “last mile” challenge pertaining to real world application of these algorithms. However, pragmatic AI trials face methodological challenges including complex statistical analysis requirements for handling time-varying confounders [48], difficulty in maintaining equipoise as algorithms update, and balancing pragmatism with sufficient measurement rigor.

In a Stepped Wedge Cluster Randomized Trial (SW-CRT), all clinical sites begin in the control phase and sequentially switch to the intervention at randomized intervals. This design isolates the true treatment effect by utilizing two simultaneous comparisons: longitudinally (comparing a site to its pre-intervention self) and concurrently (comparing active sites to control sites), effectively ruling out biases caused by temporal trends.

This design is valuable for AI evaluation as it accommodates the logistical constraints of implementing complex workflows while controlling for temporal trends and ensuring all sites eventually receive the tool.

A compelling example of SW-CRT in clinical AI is the study by Manz et al. [49], which tested machine learning–derived mortality predictions paired with behavioral nudges. The intervention significantly increased goals-of-care conversations for high-risk cancer patients, demonstrating that the outcome was not merely a function of model accuracy but rather a sociotechnical effect shaped by workflow integration, clinician behavior, and system-level context. By utilizing a stepped-wedge design, the trial effectively evaluated the interaction between the algorithm, the deployment strategy, and clinician adoption across multiple sites while controlling for temporal trends.

Clinical AI trials face four main causal inference challenges that are usually absent from conventional therapeutic RCTs and that require pre-specification of the causal estimand governing the analysis to help interpret the study results (Table 3). The estimand is the precise treatment effect that a study aims to estimate, defined in advance by specifying the target population, outcome, comparison, and how intercurrent events are handled [50].

First, predictive AI systems induce performative prediction: for instance, when a sepsis alert is generated, the clinician acts on it, and that action changes the patient’s physiological trajectory, which in turn becomes input to the next prediction cycle. This time-varying treatment–confounder feedback violates the assumptions of standard regression because conditioning on post-treatment variables (e.g., vital signs measured after the alert) introduces collider bias. Marginal structural models using inverse probability weighting (IPW) address this by creating a pseudo-population in which treatment assignment at each time point is independent of time-varying confounders, enabling unbiased estimation of the causal effect of sustained adherence to the AI-guided care [51–53].

Second, if an AI model is updated from version 1.0 to version 1.1 during a trial which can occur under governance frameworks such as the FDA’s Predetermined Change Control Plan, the same patient cannot be observed under both versions, and before–after comparisons confound the version change with secular trends. The parametric g-formula addresses this by modeling the joint distribution of time-varying covariates and outcomes under each deployment regime, simulating what outcomes would have been under continuous deployment of the original model versus the update sequence actually implemented [54]. Within the ICH E9(R1) estimand framework, the model update constitutes an intercurrent event whose handling strategy must be pre-specified [50].

Third, equity-relevant patient subgroups such as older patients, racial and ethnic minorities, patients from low- and middle-income countries are typically underpowered in any single-site trial. Naïve pooling of data across sites introduces heterogeneity bias due to differences in clinical workflows, implementation fidelity, and patient populations. Data fusion techniques provide a principled approach to combining randomized trial data with observational data from additional sites, conditioned on a shared causal model, enabling estimation of subgroup-specific treatment effects that no single data source could reliably estimate [55,56].

Fourth, a trial conducted at an academic medical center may not predict the clinical benefit of deploying the same AI system at a community hospital with a different patient population and workflow structure which constitutes the “illusory generalizability” problem documented by Chekroud et al. [38]. External validation using discrimination metrics such as the AUC assesses predictive accuracy at the target site but does not estimate the causal effect of deployment on patient outcomes at that site. Transportability analysis addresses this by reweighting trial estimates using the target site’s covariate distribution, producing a transported treatment effect estimate under formalized assumptions about effect modification across settings [57,58].

The selection of the appropriate causal inference method depends on the nature of the monitoring signal that triggered the formal evaluation: feedback-loop confounding might require the use of marginal structural models (MSMs) version transitions might be analyzed through the g-formula, equity concerns often require data fusion, and cross-site deployment is best analyzed through transportability analysis (Fig 2, Table 3).

Finally, synthetic data, digital twins, and in-silico simulations [59] are emerging tools that can complement AI evaluation frameworks without replacing real-world evidence. While these data sources must never be used to estimate primary clinical endpoints or mixed with real trial data for outcome analysis, they offer important opportunities to strengthen trial design and post-deployment safety.

Synthetic cohorts can support trial planning, including scenario-based power calculations, anticipated drift conditions, and expected enrollment patterns. Digital-twin simulations allow verification and validation stress-testing of AI models across edge cases that may be rare or difficult to capture prospectively. After model updates, synthetic data can be used for rapid regression testing to ensure that recalibration or architectural changes have not introduced performance degradation before silent-mode deployment.

These uses position synthetic data as an adjunct to adaptive and pragmatic AI trials, valuable for preparation and safety assurance, but never a substitute for prospective evaluation in real patients. These methodological advances collectively provide a toolkit for addressing the complex analytical requirements of iterative AI evaluation.

## How to ensure better care from better models

Implementing predictive AI in clinical practice should foremost improve patient care and support clinicians in making timely and accurate decisions for those who are historically marginalized. Achieving this outcome requires attention to multiple factors when evaluating AI tools.

AI tools must be transparent about what data were used to train them and who was involved in the curation and model development to promote both clinician and patient trust. Collaboration with patients and their advocates early in the design process can improve acceptance, particularly in marginalized communities [60]. If power dynamics between patients and providers is acknowledged, this approach promotes shared decision-making and acknowledges patient rights to understand and question the technology guiding their care.

Clinician engagement in designing, implementing, and evaluating the tools they will use is essential. Educational initiatives that demystify how AI works, its decision boundaries and uncertainties, and most crucially, its risk of harm, shall enable clinicians to leverage this powerful technology safely and responsibly [61]. Three educational formats offer practical starting points for institutions deploying clinical AI. First, simulation-based training can present clinicians with historical scenarios of AI performance degradation. For instance, cases in which the Epic Sepsis Model generated alerts with declining positive predictive value over a defined period, requiring participants to identify alert fatigue signals and practice structured override reasoning. Such training can be embedded within existing simulation curricula at low marginal cost [62]. Second, real-time monitoring dashboards displaying model-health indicators such as performance, process, outcomes, and fairness metrics, can be reviewed within routine clinical rounds or governance meetings, integrating AI oversight into existing quality-review workflows [63]. Third, compiling AI-assisted clinical decisions with annotated outcomes as case libraries could enable multidisciplinary retrospective review of cases in which predictions were incorrect to improve analysis of the contextual factors (patient characteristics, data quality, workflow environment) that drove those errors [64]. Education about AI tools is requisite to help healthcare personnel and patients recognize the limits of a prediction within a specific context. Calibration literacy and distributional reasoning refer to the ability to interpret model calibration metrics and to recognize that stated probabilities do not represent certainty for individual patients, particularly when the patient differs from the training population in age, comorbidity burden, care setting, or geographic context, in which case reported aggregate performance may not apply and cause biases [65]. Workflow-override judgment is the capacity to recognize automation bias and to apply structured reasoning when an AI alert conflicts with clinical assessment: specifically, weighing the relative clinical cost of a false positive against a false negative for the individual patient at that moment in their care trajectory [66].

Certainly, AI cannot foresee every complication, every disease trajectory, and every treatment response, particularly when different determinants of health are at play, from social, to epistemic, to commercial, to geopolitical. Even extensive data from under-represented groups fail to capture confounders that lead to care phenotypes, leading to spurious associations that are easily encoded in AI. Understanding these intrinsic constraints tempers expectations and underscores why AI should augment rather than replace human oversight. The limitations of AI tools should be contextualized against current clinical standards, which themselves contain inherent biases and imperfections. Notably, AI solutions need not achieve perfection but rather demonstrate incremental improvement over existing standard of care, which often lacks rigorous evaluation and may perform sub optimally in various clinical scenarios [67].

Moreover, the key performance indicators of health AI must shift from preoccupation with narrow metrics (such as area under the receiver operating characteristic curve (AUC)) to more meaningful, patient-centered and clinician-centered endpoints (improvement of clinical workflow, low number of false alarms) [68]. These key components and lifecycle of AI evaluation are presented in Table 4 and in Fig 1.

**Table 4:** Actionable Checklist for Clinical AI Operationalization. A strategic checklist for the design, governance, and evaluation of clinical AI. This “mini-playbook” outlines seven essential requirements for translating AI principles into practice, ensuring systems are safe, equitable, and integrated effectively into clinical care.

Lifecycle phase	Actionable step	Implementation focus
**1. Scoping & design**	**Define stakeholder context**	Explicitly map roles and needs for all users (patients, nurses, physicians, administrators) to ensure the tool fits the specific clinical environment.
	**Prioritize workflow integration**	Design for the complete clinical workflow rather than an isolated prediction model. Incorporate **explainability** features to foster user trust and adoption.
	**Select meaningful endpoints**	Shift focus from technical metrics (e.g., AUC) to **patient-centered outcomes** (e.g., reduced adverse events, shorter length of stay).
**2. Governance & safety**	**Pre-specify update protocols**	Establish clear governance for model updates, including specific criteria for **drift monitoring** and “circuit breakers” for pausing or rolling back deployment.
	**Plan for de-implementation**	Define objective triggers (e.g., harm signals, workflow bottlenecks) that would necessitate withdrawing the AI system.
**3. Continuous evaluation**	**Mandate equity checks**	Conduct systematic audits to ensure performance consistency across all demographic and clinical subgroups *before* and *during* deployment.
	**Monitor holistic metrics**	Track real-time metrics beyond accuracy, including **calibration** drift, **false-alarm burden** (alert fatigue), and unintended behavioral shifts in clinicians.

## Conclusion

In this narrative review, we outlined practical steps for evaluating clinical AI through adaptive trial designs, continuous post-deployment monitoring, explicit update governance, and a reorientation toward outcomes that reflect patient experience and workflow impact rather than technical accuracy alone. These approaches encourage investigators to move beyond static, one-time assessments and adopt methods capable of capturing how AI tools perform as clinical practice, data distributions, and user behaviors evolve.

The framework proposed in this review integrates performance monitoring, clinical impact monitoring, hybrid evidence generation, causal estimands, and model update governance within a unified decision pathway. It offers clinicians and trialists a structured approach to navigate the entire evaluation lifecycle of predictive AI, including post-deployment surveillance that can trigger rigorous evidence generation when indicated.

Looking ahead, clinicians should remain actively involved in designing, implementing, and monitoring AI systems to ensure they address real clinical needs and maintain patient safety [69]. Trialists should use pragmatic and adaptive approaches that accommodate model updates and capture the realities of clinical workflow. Regulators should promote lifecycle-based evaluation frameworks that require transparent reporting of model changes and support consistent post-market monitoring. Together, these efforts will help build the trustworthy, context-aware evidence base needed for the safe adoption of AI in routine clinical care.
